# Production of Magnesium Dilactate through Lactic Acid Fermentation with Magnesium Carbonate

**DOI:** 10.3390/microorganisms12102011

**Published:** 2024-10-03

**Authors:** Sangmin Won, Ho Young Kang

**Affiliations:** Department of Microbiology, Pusan National University, Busan 46241, Republic of Korea

**Keywords:** lactic acid bacteria, magnesium lactate, magnesium carbonate, fermentation

## Abstract

Magnesium dilactate is increasingly sought after for its applications in the pharmaceutical, food, and dietary supplement industries due to its essential role in various physiological processes. This study explores a sustainable method for synthesizing magnesium dilactate through lactic acid fermentation using tomato juice, coupling the neutralization of lactic acid with hydrated magnesium carbonate hydroxide. Utilizing the lactic acid bacteria *Lactobacillus paracasei* and *Lactobacillus plantarum*, fermentation was optimized in a 50% diluted MRS medium supplemented with glucose and tomato juice supplemented with glucose, yielding a maximum lactate concentration of 107 g/L. Notably, fermentation in diluted media proved more effective than in undiluted tomato juice, highlighting the inhibitory effects of certain organic compounds and the physical nature of the original tomato juice. Post-fermentation, magnesium lactate was crystallized, achieving high recovery rates of up to 95.9%. Characterization of the product through X-ray diffraction and scanning electron microscopy confirmed its crystalline purity. This research underscores the viability of tomato juice as a fermentation substrate, promoting the valorization of agricultural by-products while providing an eco-friendly alternative to traditional chemical synthesis methods for magnesium dilactate production.

## 1. Introduction

Magnesium dilactate is a compound of considerable interest due to its widespread applications in the pharmaceutical, food, and dietary supplement industries. This compound is valued for its role as a magnesium supplement, which is essential for numerous physiological processes, including enzyme function, muscle contraction, and nerve transmission [1]. Given its importance, there is a growing demand for efficient and sustainable production methods for magnesium dilactate.

Traditionally, magnesium dilactate has been synthesized through various chemical processes. These methods often involve the use of strong acids, bases, and other reactive chemicals, which can pose environmental and safety hazards [2]. Additionally, such chemical processes can be costly and generate substantial amounts of waste. As sustainability becomes an increasingly critical focus in industrial practices, there has been a notable shift towards greener and more efficient production methods.

One promising approach to address these challenges is the use of biotechnological methods for the synthesis of magnesium dilactate. Lactic acid fermentation offers a more environmentally friendly alternative. This process utilizes microorganisms to convert carbohydrates into lactic acid, which can then be used to produce magnesium dilactate. The advantages of fermentation include the use of renewable resources and the potential for lower energy consumption compared to traditional chemical synthesis [3,4,5,6].

Lactic acid fermentation has been widely studied and optimized in recent years. Advances in microbial biotechnology have led to the development of engineered strains with enhanced lactic acid production capabilities. For instance, researchers have genetically modified strains of *Lactobacillus* and other bacteria to improve their metabolic pathways, resulting in higher yields of lactic acid [7]. The optimization of fermentation conditions, such as temperature, pH, and substrate concentration, has also been shown to significantly impact the efficiency of lactic acid production [8,9,10,11].

Following the production of lactic acid through fermentation, the next critical step in the synthesis of magnesium dilactate involves the neutralization of lactic acid with a suitable magnesium compound. Hydrated magnesium carbonate hydroxide has emerged as a preferred neutralizing agent due to its ability to provide a controlled and efficient reaction environment [12,13]. This compound helps to form magnesium dilactate while minimizing the formation of impurities and by-products.

The choice of neutralizing agent is crucial for optimizing the yield and purity of magnesium dilactate. Hydrated magnesium carbonate hydroxide, in particular, has been shown to offer several advantages over traditional neutralizers. Studies indicate that it provides a more controlled reaction and results in fewer side reactions, which contributes to the overall efficiency of the synthesis process. The use of this neutralizing agent aligns with the principles of green chemistry, which emphasize reducing the use of hazardous substances and minimizing environmental impacts.

Integrating lactic acid fermentation with the use of hydrated magnesium carbonate hydroxide for neutralization represents a significant advancement in the production of magnesium dilactate. This combined approach not only enhances the sustainability of the production process but also improves the economic feasibility of manufacturing magnesium dilactate. By reducing the reliance on harsh chemicals and minimizing waste, this method offers an eco-friendlier alternative to traditional synthesis routes.

This research aims to investigate the synthesis of magnesium dilactate using a combined approach of lactic acid fermentation and neutralization with hydrated magnesium carbonate hydroxide using tomato juices to maximize lactic acid production and evaluate the effectiveness of hydrated magnesium carbonate hydroxide in producing magnesium dilactate. The outcomes of this research could play a pivotal role in advancing sustainable production practices in the pharmaceutical and food industries.

## 2. Materials and Methods

### 2.1. Fermentation Process

#### 2.1.1. Microorganisms

Two lactic acid bacteria, *Lactobacillus paracasei* and *Lactobacillus plantarum*, were applied in this study. *Lactobacillus paracasei* (KCTC 13169) was obtained from the Korean Collection for Type Cultures (Daejeon, Korea). *Lactobacillus plantarum* was isolated from regular Korean yogurt through screening on MRS agar and defined via 16S-rRNA sequencing [14]. All three cultures were subcultured continuously on MRS agar plates to maintain for use in further experiments. Before the start of each experiment, each bacterial culture was precultured in MRS medium to the stationary phase and was used as the inoculum for the fermentation experiment.

#### 2.1.2. Culture Medium

The MRS medium (the specific medium for lactic acid bacteria) was used to screen suitable bacteria for fermentation. Then, selected bacterial strains were used for tomato juice fermentation. The cultivation was also carried out with a 50%-diluted MRS medium and a 50%-diluted tomato juice to investigate the feasibility of saving materials.

#### 2.1.3. Raw Materials

Tomato juice was obtained as the fluid derived from tomato seed harvesting processes. They were collected and kept frozen at −18 °C for continuous use for subsequent experiments. The juice was introduced to determine pH and organic acid and sugar contents. The juice was pasteurized and filtered to remove particulate matter. Glucose (the substrate for fermentation) was additionally added at 100 g/L in the fermentation process.

High-purity hydromagnesite, also known as hydrated magnesium carbonate (HMC), is derived from magnesium extracted from bittern or brine sources HMC, with a purity greater than 99%, was sourced from EcoMag Ltd., located in Chatswood, NSW, Australia. Before being used for the neutralization of the fermentation, the fine HMC powder was mixed with water in a 1:3 weight-to-weight ratio to create a slurry.

#### 2.1.4. Culture Condition

The lab-scale fermentation experiment was carried out in a 500 mL media bottle with a 200 mL working volume and incubated in a shaking incubator at 37 °C. The pH of the fermentation media was monitored using a calibrated pH meter. The organic acids and sugar contents were determined via high-performance liquid chromatography (HPLC) using a Dionex Ultimate3000 high-performance liquid chromatograph (ThermoFisher Scientific Inc., Waltham, MA, USA) [15]. The HPLC conditions are summarized in Table 1.

In the first experiment, the fermentation was carried out with both *L. plantarum* and *L. paracasei* to look for the better bacterial strain that can perform fermentation glucose coupling with the synthesis of magnesium lactate. *L. paracasei* was then selected for the subsequent experiments with diluted MRS medium and tomato juice to investigate the feasibility of saving materials. All of the experimental set-ups are summarized in Table 2. Glucose (100 g/L) was added to each medium at the beginning, whereas HMC (10 g), slurried with water to a weight of 40 g, was added into each medium at an interval of 12 h.

### 2.2. Magnesium Lactate Crystallization and Recovery

At the end of the fermentation experiment, the fermented media were filtered to remove suspended solids and biomass before their introduction to the crystallization process. The solution was heated during stirring to enhance evaporation and concentrate the solute up to the crystallization point. The crystallized magnesium lactate was isolated from the liquor via vacuum filtration using a Buchner funnel with filter paper. The crystals were subsequently washed with alcohol 99.9% to remove any residual impurities and unreacted lactic acid. The washing procedure was repeated until the wash filtrate was clear, indicating thorough purification. The washed crystals were subjected to drying in an oven at 60 °C for 24 h to remove residual moisture. This step was crucial to prevent the formation of hydrate phases and ensure the stability of the magnesium lactate crystals [16].

### 2.3. Product Characterization and Analytical Techniques

#### 2.3.1. X-ray Fluorescence (XRF) Analysis

The obtained products were also subjected to a Wavelength-Dispersive X-ray Fluorescence Spectrometer (WD-XRF; S-8 Tiger, Bruker, Billerica, MA, USA.) using the fused glass method at the Daegu Center of the Korea Basic Science Institute (KBSI). X-ray fluorescence spectroscopy was employed to determine the elemental composition of the magnesium lactate crystals. This technique involves irradiating the sample with X-rays, causing the elements within the sample to emit secondary (or fluorescent) X-rays. These emitted X-rays are characteristic of the elements present and are detected to identify and quantify the elemental composition [17]. The dried magnesium lactate crystals were analyzed using an XRF spectrometer. The analysis was performed under optimized conditions to ensure minimal interference and accurate detection of magnesium and other trace elements. The spectra were processed to determine the relative concentrations of magnesium, calcium, and other relevant elements.

#### 2.3.2. X-ray Diffraction (XRD) Analysis

The dry Mg lactate product was subjected to an X-ray diffractometer (Xpert 3 Powder, Malvern Panalytical, UK) at the Core Research Facilities of Pusan National University to determine the crystal phase. X-ray diffraction was utilized to elucidate the crystalline structure of the magnesium lactate. XRD involves the diffraction of X-rays by the crystal lattice, providing information on the unit cell dimensions and crystallinity of the sample [18]. The magnesium lactate crystals were analyzed using a powder X-ray diffractometer. The diffraction patterns were collected over a range of 2θ angles and compared with standard diffraction databases to identify the crystalline phases present. Peak positions and intensities were used to verify the crystallinity and phase purity of the magnesium lactate.

### 2.4. Scanning Electron Microscopy (SEM) Analysis

The morphology of magnesium lactate was characterized using scanning electron microscopy (SEM). Prior to analysis, the magnesium lactate samples were prepared by placing a small quantity on a conductive carbon tape affixed to an aluminum stub. The samples were then coated with a thin layer of gold using a sputter coater to enhance conductivity and minimize charging effects during imaging.

SEM imaging was conducted using a JEOL JSM-IT100 instrument operated at an accelerating voltage of 15 kV. Images were captured at various magnifications to provide a comprehensive view of the particle morphology and size distribution.

### 2.5. Statistical Analysis

All of the experimental procedures were performed in triplicate to ensure accuracy and reproducibility. Data were expressed as mean ± standard deviation. Statistical significance was determined using one-way ANOVA followed by Tukey’s post hoc test. A *p*-value of less than 0.05 was considered statistically significant.

## 3. Results and Discussion

### 3.1. Characteristics of Tomato Juice Used for Fermentation Process

Tomato juice offers distinct compositional attributes that impact its suitability for lactic acid fermentation with lactic acid bacteria (LAB). Tomato juice, with its high water content, low sugar levels (14.64 g/L of fructose and 13.12 g/L of glucose), and mild acidity (pH 4.5–5.0), provides a favorable environment that supports LAB growth for fermentation. This initial nature of tomato juice ensures a favorable environment for LAB proliferation and lactic acid production, avoiding issues related to excessive acidity or low nutrient availability. Before fermentation, lactic acid and acetic acid were not detected in the tomato juice. Besides sugars, such as fructose and glucose, tomato juice also contains some organic acids, such as citric acid, glutamic acid, tartaric acid, malic acid, and ascorbic acid, with their concentrations presented in Table 3.

### 3.2. Production of Magnesium Lactate through Fermentation Process

**Figure 1:**
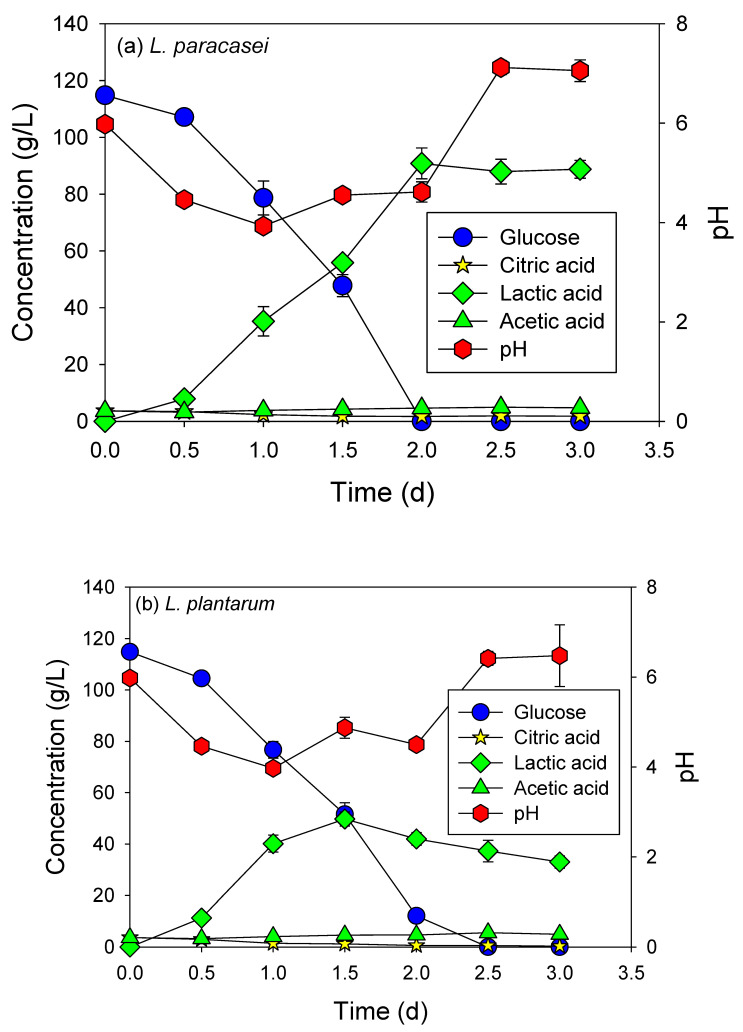
Fermentation of glucose added with HMC in MRS medium using (**a**) *L. paracasei* and (**b**) *L. plantarum*.

In the first experiment, both *Lactobacillus paracasei* and *Lactobacillus plantarum* successfully demonstrated the possibility of synthesizing magnesium lactate through fermentation in an MRS medium added to glucose and HMC. However, *L. paracasei* was indicated as being a better option for the further fermentation experiment (Figure 1). Glucose was consumed within 2 days, resulting in the production of 95 g/L of lactate when *L. paracasei* was used. In contrast, *L. plantarum* consumed glucose over a period of 2.5 days, and 52 g/L of lactate was produced. Citric acid and acetic acid concentrations were stable at a steady state during fermentation. The pH of the medium continuously decreased during the first stage, complying with the production of lactic acid. However, the pH increased at the end of the experiment owing to the consumption of lactic acid by the magnesium carbonate reaction to produce magnesium lactate.

**Figure 2:**
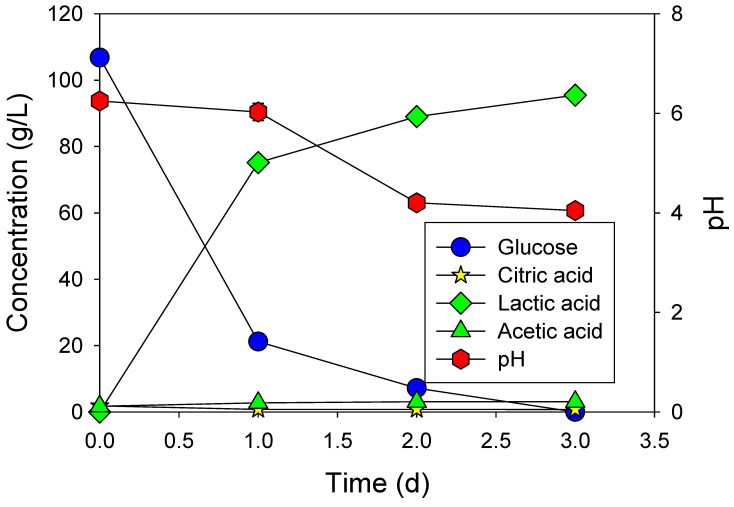
Fermentation of glucose added with HMC in 50% diluted MRS medium using *L. paracasei*.

Following the selection of suitable bacteria, fermentation was conducted using a 50% diluted MRS medium supplemented with glucose. The results indicated that glucose consumption extended over a period of 3 days. Nevertheless, a high concentration of lactate was achieved, reaching 107 g/L (Figure 2).

**Figure 3:**
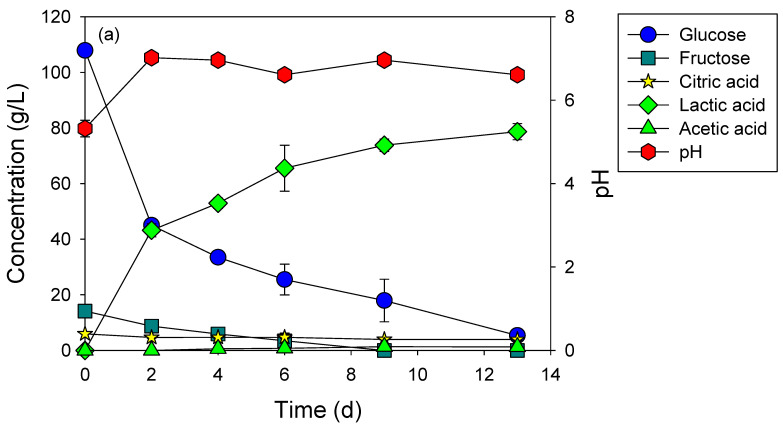

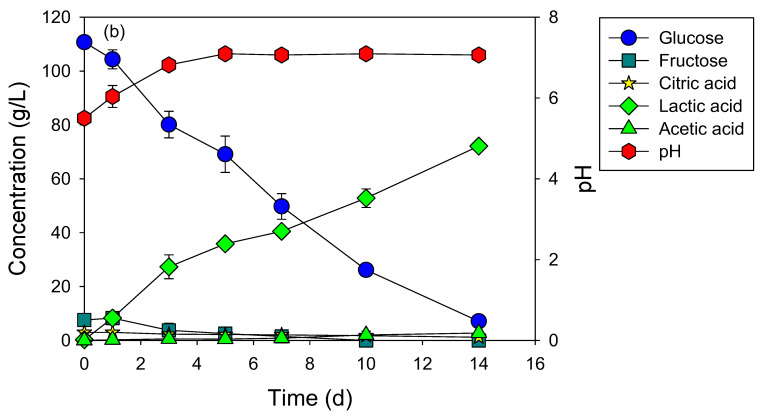
Fermentation of glucose added with HMC in original tomato juice (**a**) and 50% diluted tomato juice media (**b**) using *L. paracasei*.

The fermentation of glucose supplemented with HMC was conducted using *Lactobacillus paracasei* in both undiluted and 50% diluted tomato juice media, as shown in Figure 3. In the case of 100% tomato juice, the fermentation process extended over 13 days, yielding 72 g/L of magnesium lactate. Conversely, when utilizing 50% diluted tomato juice, the fermentation duration increased to 14 days, resulting in a higher production of 78 g/L of lactate. This indicates that fermentation in 50% diluted tomato juice is more effective than in the undiluted medium. This enhanced efficacy may be attributed to the presence of inhibitory substances, such as organic acids, found in tomato juice, which could impede fermentation in the undiluted condition. The minor amount of fructose originally present in the tomato juice was also consumed simultaneously with the consumption of glucose by lactic acid bacteria to produce lactic acid (Figure 3).

### 3.3. Magnesium Lactate Recovery through Crystallization

**Figure 4:**
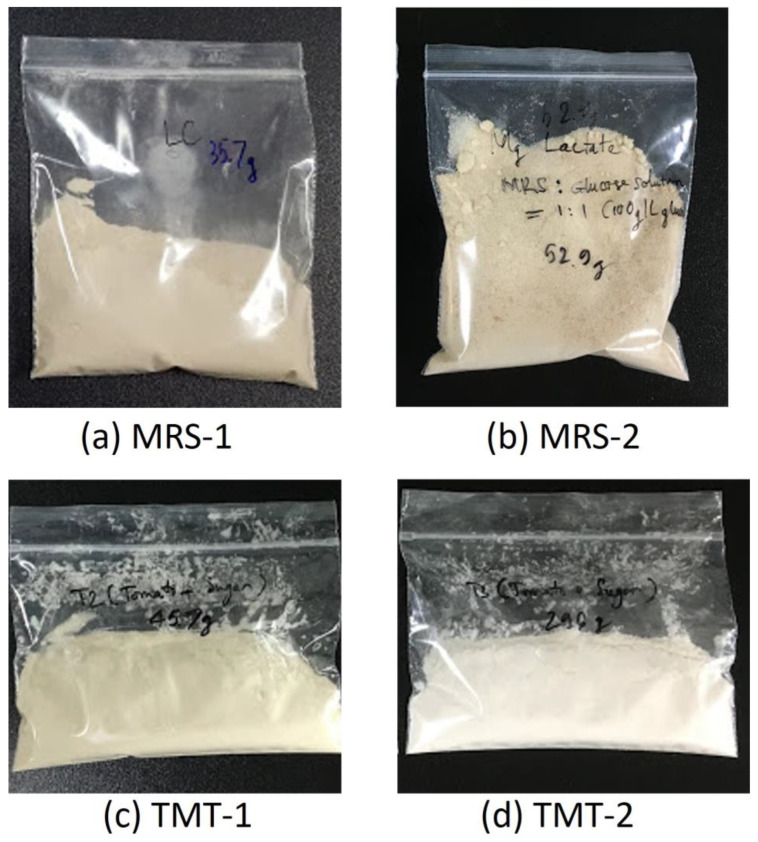
(**a**–**d**) Products obtained from the crystallization of magnesium lactate produced via fermentation.

The product obtained from the crystallization of magnesium lactate produced through fermentation is shown in Figure 4. After drying overnight in the oven, magnesium lactate was recovered as a fine powder. A darker color was observed with the magnesium lactate product obtained from the fermentation using MSR medium (Figure 4a,b). This color came from the dark color of MRS medium. Similarly, the yellowish color of the product obtained from the fermentation with tomato juice was from the yellow color of the tomato juice (Figure 4c,d). At commercial scale, the color of a product can be removed via a washing stage. In the MRS-1 trial, a final lactate concentration of 95 g/L was achieved, resulting in a yield of 0.9 g/g and a magnesium lactate recovery rate of 79.9%. The MRS-2 trial yielded the highest concentration, reaching 107 g/L, with a yield of 0.92 g/g and an impressive recovery rate of 95.9%. With the fermentation carried out using tomato juice, the TMT-1 trial produced a final concentration of 72 g/L, yielding 0.63 g/g and a recovery rate of 82.4%. The TMT-2 trial showed a slight improvement, with a concentration of 78 g/L, a yield of 0.73 g/g, and a recovery rate of 91.1% (Table 4). Therefore, the high concentration and yield of magnesium lactate recovery through crystallization can be obtained using 50% diluted tomato juice medium using L. paracasei.

### 3.4. Characterization of Magnesium Lactate Products

**Figure 5:**
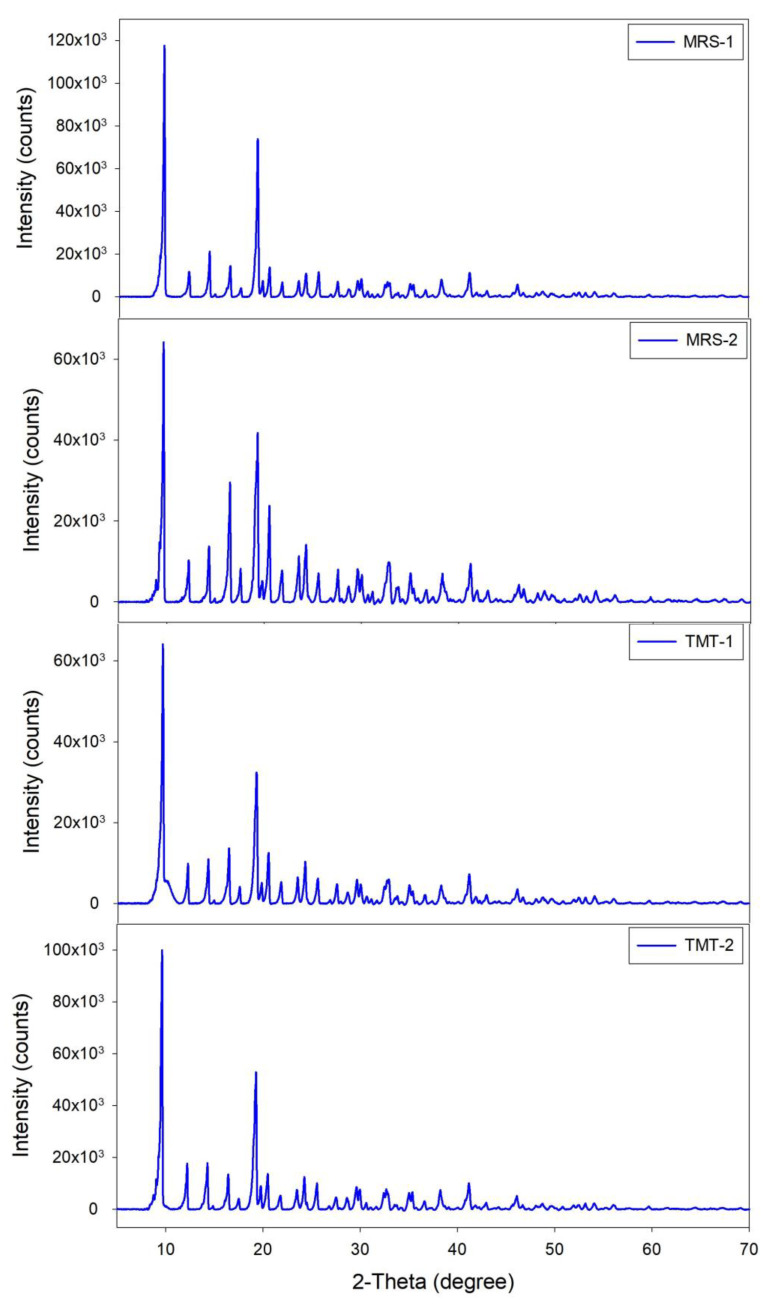
XRD patterns of four products obtained through fermentation.

The X-ray diffraction (XRD) analysis of the four products obtained through fermentation demonstrated identical diffraction patterns across all samples (Figure 5). The results revealed a series of well-defined peaks, indicating a crystalline structure characteristic of magnesium lactate. The most prominent peaks appeared at specific 2θ values, consistent with the standard diffraction pattern for magnesium lactate, suggesting that the fermentation process effectively yielded a pure crystalline product. The peak intensities were similar, reflecting uniformity in crystallinity among the samples. The consistent XRD patterns across all four products confirm the reproducibility of the fermentation process and the successful synthesis of magnesium lactate.

The X-ray fluorescence (XRF) analysis of the magnesium lactate product provided detailed insights into the elemental composition of the final product (Table 5). Notably, magnesium was identified as the predominant element, constituting a significant percentage of the total composition, which underscores the successful synthesis of magnesium lactate. Additionally, trace amounts of other elements were found, likely stemming from impurities during the synthesis process. Specifically, magnesium accounted for approximately 6.69–10.17% of the overall elemental content, while trace elements were present at minimal levels of the total composition.

These findings validate the anticipated elemental profile of magnesium lactate, indicating that the employed synthesis method effectively concentrated the desired magnesium component while limiting contamination from extraneous elements. Such information is essential for assessing the purity and potential applications of magnesium lactate in various sectors, including food, pharmaceutical, and agriculture applications.

**Figure 6:**
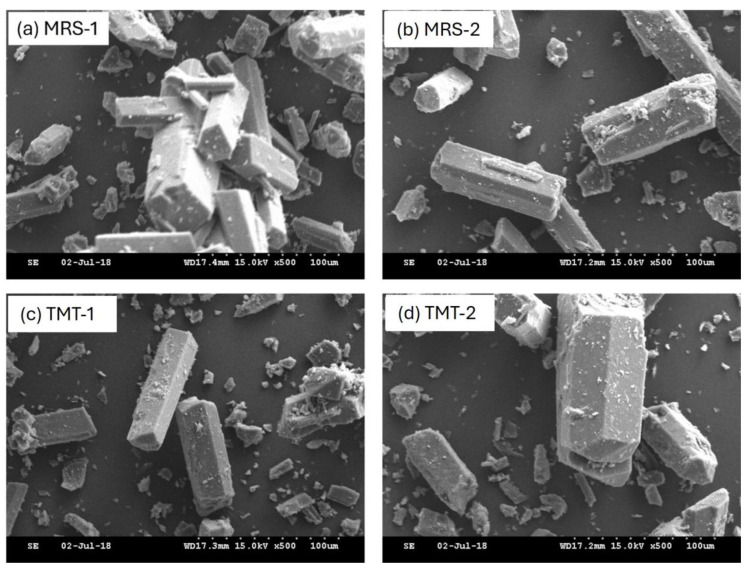
SEM images of products obtained through fermentation at magnification of 500×.

The scanning electron microscopy (SEM) analysis of the magnesium lactate product revealed consistent morphological characteristics across all samples examined (Figure 6). The surface texture of the magnesium lactate particles was smooth, with distinct features indicating crystalline structures. This crystalline morphology is indicative of the successful synthesis and formation of magnesium lactate, aligning with the expected characteristics reported in the literature. Overall, the SEM analysis confirmed that the morphology of the magnesium lactate samples was consistent.

We successfully produced magnesium lactate via the fermentation of tomato juice with magnesium carbonate. This underscores the feasibility of using unconventional substrates in industrial fermentation while promoting sustainability. When compared with previous studies, our findings show promising potential for cost-effective and environmentally friendly production methods. This study could pave the way for further exploration into agro-based fermentation processes and their applications in various industries, from nutrition to waste management. This study also stands out by successfully using magnesium carbonate, a relatively cost-effective and stable form of magnesium, which was also recovered from a waste stream of the salt production industry. This is notable because magnesium carbonate is not as commonly used due to its lower solubility compared to other magnesium salts. The use of tomato juice, especially when it comes from processing by-products, contributes to a circular economy by reducing food waste and utilizing renewable resources. This is an attractive feature in the modern push toward sustainable industrial processes. If this process is scalable, it could offer a new avenue for industrial production of magnesium lactate, particularly in regions with ample tomato production. This could benefit both the agricultural and biochemical industries, creating a synergy between food production and chemical synthesis. However, more extensive studies are required before the scaling-up of this technology because the current product is not favorable color. More studies on the purification of products are required before this process is commercially available at an industrial scale.

## 4. Conclusions

This study demonstrates the effective production of magnesium lactate through the fermentation of tomato juice using *Lactobacillus paracasei*. The juice’s high water content, moderate acidity, and low sugar levels create an optimal environment for lactic acid bacteria, facilitating efficient fermentation. *Lactobacillus paracasei* achieved a maximum lactate concentration of 107 g/L, outperforming *Lactobacillus plantarum*. Notably, fermentation in a 50% diluted tomato juice medium proved more effective than in undiluted juice, suggesting that organic acids in undiluted juice may inhibit the process. Crystallization yielded high recovery rates, particularly with diluted juice, and characterization via X-ray diffraction and scanning electron microscopy confirmed the purity and crystalline structure of the magnesium lactate produced. This research highlights the potential of tomato juice as a fermentation substrate and suggests opportunities for valorizing agricultural by-products, with implications for industrial applications in food, pharmaceuticals, and agriculture.

## Figures and Tables

**Table 1:** HPLC conditions for analyzing organic acids and sugars.

	Sugars	Organic Acid
Column	YMC-Pack Polyamine II (250 × 4.6 mm)	YMC-Triart C18 (3 µm, 12 nm), 150 × 3.0 mm
Eluent	Acetonitrile/water (75/25, *v*/*v*)	20 mM H_3_PO_4_
Oven temp.	26 °C	37 °C
Flow rate	1.0 mL/min	1.0 mL/min
Detector	RI (Shodex RI-101, Tokyo, Japan)	UV at 220 nm
Inj. Volume	20 ㎕	20 ㎕

**Table 2:** Experimental set-up.

Name	Medium	Bacteria	Note
MRS-0	MRS medium	*L. plantarum*	To test performance of two bacteria and select the suitable bacteria for following experiments
MRS-1	MRS medium	*L. paracasei*	
MRS-2	MRS medium diluted with water at 1:1 ratio	*L. paracasei*	To test the fermentation performance with saving materials
TMT-1	Tomato juice	*L. paracasei*	To test the fermentation performance with real tomato juice
TMT-2	Tomato juice diluted with water at 1:1 ratio	*L. paracasei*	To test the fermentation performance with diluted tomato juice

**Table 3:** Sugar and organic acid content in tomato juice used for this experiment.

**Fructose (g/L)**	**Glucose (g/L)**	**Citric Acid (g/L)**	**Glutamic Acid (g/L)**	**Tartaric Acid (mg/L)**
14.64 ± 0.17	13.12 ± 0.22	6.42 ± 0.18	1.24 ± 0.10	596.66 ± 7.25
**Malic Acid (mg/L)**	**Ascorbic Acid (mg/L)**	**Succinic Acid (mg/L)**	**Lactic Acid (mg/L)**	**Acetic Acid (mg/L)**
714.36 ± 9.85	3.93 ± 0.44	ND *	ND	ND

* ND: non-detected.

**Table 4:** Product recovery evaluation.

Test Name	Final Lactate Concentration (g/L)	Lactic Acid Yield from Glucose (g/g)	Magnesium Lactate Recovery from Solution (%)
MRS-1	95	0.9	79.9
MRS-2	107	0.92	95.9
TMT-1	72	0.63	82.4
TMT-2	78	0.73	91.1

**Table 5:** Percentage of the detectable element in the obtained products by XRF.

Sample	Al_2_O_3_ (%)	CaO (%)	Cr_2_O_3_ (%)	Fe_2_O_3_ (%)	K_2_O (%)	MgO (%)	MnO (%)	Na_2_O (%)	P_2_O_5_ (%)	LOI (%)
	Al (%)	Ca (%)	Cr (%)	Fe (%)	K (%)	Mg (%)	Mn (%)	Na (%)	P (%)	
MRS-1	0.09	0.11	1.99	4.34	0.11	11.09	0.12	0.06	0.44	81.65
	0.02	0.08	0.68	1.52	0.05	6.69	0.09	0.02	0.10	
MRS-2	0.08	0.02	0.55	1.16	0	15.93	0.03	0.03	0	82.21
	0.02	0.01	0.19	0.40	0	9.61	0.02	0.01	0	
TMT-1	0.08	0.03	0.59	1.24	0.04	16.87	0.03	0.07	0.06	80.99
	0.02	0.02	0.20	0.43	0.02	10.17	0.02	0.03	0.01	
TMT-2	0.09	0.02	0.74	1.54	0	16.03	0.04	0.04	0.01	81.50
	0.02	0.01	0.25	0.54	0	9.67	0.03	0.01	0.00	
Mg lactate dihydrate (theoretical)	-	-	-	-	-	16.90	-	-	-	83.10
	-	-	-	-	-	10.19	-	-	-	
Mg lactate anhydrous (theoretical)	-	-	-	-	-	19.91	-	-	-	80.09
	-	-	-	-	-	12.01	-	-	-	

Highlight numbers indicated the content of Mg which is the basic data for comparison and determination of Mg lactate forms.

## Data Availability

The raw data supporting the conclusions of this article will be made available by the authors on request.
